# Anxiety among Patients Visiting for Periodontal Therapy in a Tertiary Care Dental Hospital: A Descriptive Cross-sectional Study

**DOI:** 10.31729/jnma.6109

**Published:** 2021-07-31

**Authors:** Bhagabat Bhattarai, Sujaya Gupta, Sirjana Dahal, Deepak Kumar Roy, Saroj Pant, Rachana Karki, Tanu Thakuri

**Affiliations:** 1 Department of Periodontics and Oral Implantology, Kathmandu Medical College, Bhaktapur, Nepal; 2 Dentistry, Institute of Medicine, Maharajgunj, Kathmandu, Nepal; 3 Department of Conservative Dentistry and Endodontics, Kathmandu Medical College, Bhaktapur, Nepal

**Keywords:** *bone drilling*, *local anaesthetics*, *Nepal*, *scaling*, *tooth drilling*

## Abstract

**Introduction:**

The aetiological factors of dental fear include negative information, witnessing or having a bad experience, and negative conditions related to periodontal treatment. Modified Dental Anxiety Scale Nepali version, is one of the tools used in epidemiological studies to measure dental anxiety in adults. The objective was to find out the prevalence of anxiety among dental patients visiting for periodontal therapy in a tertiary care hospital.

**Methods:**

This descriptive cross-sectional study was conducted among patients visiting for periodontal therapy from November 2020 to January 2021 at a tertiary care dental hospital. Ethical clearance from Institutional Review Committee (Reference no. 0311202001) was taken before the study. Convenient sampling was done. A standard questionnaire for dental anxiety was used for data collection after receiving informed consent from the participants. Data were entered and analyzed in Microsoft Excel Sheet. Descriptive data are presented as means, standard deviations, frequencies, and percentages.

**Results:**

Among a total of 311 participants visiting for periodontal therapy, 297 (95.49%) (92.57-97.42 at 95% Confidence Interval) were having anxiety. Among total patients, 113 (36.33%) were fairly anxious, 111 (35.69%) were slightly anxious, 62 (19.94%) very anxious and 11 (3.54%) were extremely anxious. Majority of males 54 (17.36%) were slightly anxious while most females 67 (21.54%) were fairly anxious. Mean Modified Dental Anxiety Scale-Nepali score of all the participants was 11.59±3.808. Extreme dental anxiety was observed in 11 (3.54%) participants 7 (2.25%) females; 4 (1.29%) males).

**Conclusions:**

The prevalence of anxiety among patients visiting for periodontal therapy in this study was found to be higher compared to other study done in similar setting.

## INTRODUCTION

Dental anxiety persists among patients despite increased awareness about preventive approaches to oral diseases and innovations in pain reduction.^1,2^ Studies have shown 13% of patients in dental college and 20.90% in dental clinics were severely anxious.^3^ A telephone survey showed that 11.6% had high dental anxiety with female being more anxious.^4^ This was similar to some studies^5,6^ but not all.^7^

The aetiological factors of dental fear include negative information, witnessing or having bad experience and negative condition related to periodontal treatment.^8^ Lack of adequate dental health education may result in a high level of dental anxiety.^9^ Although none of existing psychometric tests are regarded as gold standard, Corah's Dental Anxiety Scale (DAS), Modified Dental Anxiety Scale (MDAS), and Kleinknecht's Dental Fear Survey are most commonly used to measure dental anxiety in adults.^10^

The objective was to find out the prevalence of anxiety among patients visiting for periodontal treatment in a tertiary care hospital.

## METHODS

This descriptive cross-sectional study was conducted in the Kathmandu Medical College (KMC), Teaching Hospital, Nepal. The data were collected from outpatient dental departments of both Kathmandu and Bhaktapur hospitals of KMC from November 2020 to January 2021 after obtaining ethical clearance from institutional review committee of KMC (Ref. 0311202001). Patients visiting for periodontal therapy were requested to participate in the study by convenience sampling. A standard questionnaire was taken from Giri, et al.^11^ study which was distributed among all the patients intended for periodontal therapy and after showing their willingness for participation in the study. An informed consent was obtained from the participants before they were given the questionnaire. The questionnaire contained questions about all the possible dental procedures. Patients not willing for periodontal therapy and under psychiatric consultation or under psychiatric medication were excluded from the study. Convenient sampling was done. A sample size of 311 was calculated by using following formula:

n = Z^2^ × p × q / e^2^

  = (1.96)^2^ × (0.74) × (1-0.74) / (0.05)^2^

  = 295.65

Where,

n = sample size
Z = 1.96 at 95% Confidence Interval (CI)
p = prevalence of dental anxiety, 74%^8^
q = 1-p
e = margin of error, 5%

Adding 5% of non-response rate, total sample was 310.43 =311. Data were entered and analysed in Microsoft Excel sheet. Descriptive findings are presented as means, standard deviations, frequencies, and percentages.

## RESULTS

Among total 311 participants, 297 (95.49%) (92.5797.42 at 95% Confidence Interval) were having anxiety. Among the total 311 participants, 113 (36.33%) were fairly anxious and 11 1 (35.69%) were slightly anxious (Table 1).

**Table 1:** Responses to Nepali version of the modified dental anxiety scale, n (%).

Questions	Not anxious n (%)	Slightly anxious n (%)	Fairly anxious n (%)	Very anxious n (%)	Extremely anxious n (%)	Mean±SD
If you went to your dentist for treatment tomorrow, how would you feel?	85 (27.33)	137 (44.05)	71 (22.83)	14 (4.50)	4 (1.29)	2.08±0.890
If you were sitting in the waiting room (waiting for treatment), how would you feel?	113 (36.33)	117 (37.62)	70 (22.51)	11 (3.54)	-	1.93±0.853
If you were about to have a tooth drilled, how would you feel?	30 (9.65)	116 (37.30)	84 (27.01)	60 (19.29)	21 (6.75)	2.76±1.081
If you were about to have your teeth scaled and polished, how would you feel?	124 (39.87)	119 (38.26)	43 (13.83)	20 (6.43)	5 (1.61)	1.92±0.967
If you were about to have a local anaesthetic injection in your gum, above an upper back tooth, how would you feel?	34 (10.93)	96 (30.87)	81 (26.05)	69 (22.19)	31 (9.97)	2.89±1.166
Total	14 (4.50)	111 (35.69)	113 (36.33)	62 (19.94)	11 (3.54)	

MDAS-N score = 11.59±3.808; minimum = 5; maximum = 21; SEM = 0.216

Among males, 54 (17.36%) were slightly anxious while 67 (21.54%) of females were fairly anxious (Table 2).

**Table 2:** Distribution of sex and age with MDAS-N scores, n (%).

MDAS scores	Not anxious (0-5) n (%)	Slightly anxious (6-10) n (%)	Fairly anxious (11-14) n (%)	Very anxious (15-18) n (%)	Extremely anxious (19-25) n (%)	Total n (%)
**Sex**						
Male	9 (2.89)	54 (17.36)	46 (14.79)	30 (9.65)	4 (1.29)	143 (45.98)
Female	5 (1.61)	57 (18.33)	67 (21.54)	32 (10.29)	7 (2.25)	168 (54.02)
**Age categories (years)**						
0-19	1 (0.32)	8 (2.57)	13 (4.18)	5 (1.61)	3 (0.96)	30 (9.65)
20-34	4 (1.29)	43 (13.83)	50 (16.08)	29 (9.32)	3 (0.96)	129 (41.48)
35-49	4 (1.29)	35 (11.25)	29 (9.32)	13 (4.18)	2 (0.64)	83 (26.69)
50-64	4 (1.29)	12 (3.86)	14 (4.50)	12 (3.86)	2 (0.64)	44 (14.15)
≥65	1 (0.32)	13 (4.18)	7 (2.25)	3 (0.96)	1 (0.32)	25 (8.04)
**Total**	14 (4.50)	11 1 (35.69)	113 (36.33)	62 (19.94)	11 (3.54)	311

The mean MDAS-N score of all the participants was The mean MDAS-N score of all the participants was 11.59±3.808 (SEM = 0.216; minimum = 5; maximum = 21, Table 3).

**Table 3:** Distribution of sex and age with cut-off value 19 for extreme anxiety, n (%).

Anxiety categories	Moderate to high (≤18 MDAS-N)	Extreme(≥19 MDAS-N)	Mean±SD	n (%)
**Sex**				
Male	139 (44.69)	4 (1.29)	11.32±3.957	143 (45.98)
Female	161 (51.77)	7 (2.25)	11.82±3.673	168 (54.02)
**Age categories (years)**				
0-19	27 (8.68)	3 (0.96)	12.43±3.830	30 (9.65)
20-34	126 (40.51)	3 (0.96)	11.91±3.596	129 (41.48)
35-49	81 (26.05)	2 (0.64)	10.96±3.830	83 (26.69)
50-64	42 (13.50)	2 (0.64)	11.93±4.201	44 (14.15)
≥65	24 (7.72)	1 (0.32)	10.40±3.797	25 (8.04)
**Total**	300 (96.46)	11 (3.54)	11.59±3.808	311

A cut-off value of 19 and above has been determined empirically^4^ to indicate extreme dental anxiety that may require special attention. Extreme dental anxiety (MDAS-N >19) was observed in 11 (3.54%) of the 311 participants (Table 3). Among them, 7 (2.25%) females and 4 (1.29%) males had extreme anxiety level (Table 3). At the threshold value of 19 and above, a dental professional should consider using additional management approaches like relaxation, systematic desensitisation or adjunctive pharmacotherapy.

The youngest age group of 0-19 years had highest mean score of 12.43 ±3.830 (Table 3). The mean MDAS-N score was found highest (11.83 ±3.774) among bachelors and above education group, but the difference was minimal (Table 4). The majority of the patients (1 18, 37.94%) had an education of bachelors and above (Table 4). Regarding occupation, most of the participants (75, 24.12%) were students followed by homemaker (68, 21.86%) and MDAS-N score, was also highest (12.51 ± 3.663) in the student group, (Table 4). The “Others” category of occupation included: artist (1), engineer (1), NGO worker (1), policeman (1), reporter (1), sportsman (1), and writer (1).

**Table 4:** Anxiety levels according to occupation and educational background, n (%).

Anxiety categories/MDAS-N scores	Not anxious (0-5)	Slightly anxious (6-10)	Fairly anxious (11-14)	Very anxious (15-18)	Extremely anxious (19-25)	Mean±SD	Total
**Education**							
Grade 0-5	1 (0.32)	21 (6.75)	23 (7.40)	12 (3.86)	1 (0.32)	11.69±3.619	58 (18.65)
Grade 6-10	3 (0.96)	24 (7.72)	16 (5.14)	10 (3.22)	5 (1.61)	11.41±4.329	58 (18.65)
Grade 11-12	6 (1.93)	16 (5.14)	26 (8.36)	14 (4.50)	1 (0.32)	11.46±3.805	63 (20.26)
Bachelors and above	4 (1.29)	42 (13.50)	42 (13.50)	26 (8.36)	4 (1.29)	11.83±3.774	118 (37.94)
Not responded	-	8 (2.57)	6 (1.93)	-	-	10.43±2.593	14 (4.50)
**Occupation**							
Banking/finance	-	3 (0.96)	3 (0.96)	-	-	10.50±2.258	6 (1.93)
Business	2 (0.64)	8 (2.57)	10 (3.22)	6 (1.93)	-	11.65±3.919	26 (8.36)
Farming/agriculture	2 (0.64)	6 (1.93)	-	3 (0.96)	-	9.27±4.519	11 (3.54)
Health profession	-	7 (2.25)	9 (2.89)	4 (1.29)	1 (0.32)	12±3.834	21 (6.75)
Homemaker	-	21 (6.75)	29 (9.32)	15 (4.82)	3 (0.96)	12.19±3.707	68 (21.86)
Manual/skilled worker	-	5 (1.61)	3 (0.96)	1 (0.32)	1 (0.32)	11.40±4.671	10 (3.22)
Office worker/Service/Job	4 (1.29)	12 (3.86)	10 (3.22)	5 (1.61)		10.42±3.854	31 (9.97)
Student	2 (0.64)	21 (6.75)	30 (9.65)	18 (5.79)	4 (1.29)	12.51±3.663	75 (24.12)
Teacher	1 (0.32)	4 (1.29)	2 (0.64)	2 (0.64)	2 (0.64)	12.36±4.523	11 (3.54)
Retired	1 (0.32)	6 (1.93)	2 (0.64)	2 (0.64)	-	10.09±3.534	11 (3.54)
Unemployed	2 (0.64)	7 (2.25)	5 (1.61)	3 (0.96)	-	10±3.905	17 (5.47)
Others	-	3 (0.96)	3 (0.96)	1 (0.32)	-	11.29±3.147	7 (2.25)
Not responded	-	8 (2.57)	7 (2.25)	2 (0.64)	-	10.82±2.675	17 (5.47)

About demographics, the mean age of the participants was 37.40± 16.208 years (SEM = 0.919) which ranged from nine years to 90 years and there were 143 (45.98%) males and 168 (54.02%) females. Since the study was conducted in Bagmati province, almost all the participants (289, 92.93%) were from Bagmati province and eight (2.57%) did not respond.

## DISCUSSION

Dental anxiety can be predicted by cancellation of dental appointment(s), memorisation of poor dental practice, gender, and age.^5^ Dental anxiety also varies with age, sex, and education.^12^ The MDAS is the modification of the Corah's Dental Anxiety Scale, which also includes question about local anaesthetic injection. Levin, et al. have reported that patients suffering from chronic periodontitis had higher anxiety compared to control group patients.^13^

The mean MDAS-N score of all participants was 11.59±3.808, this was similar to that reported by Faisal et al. (10.24±4.7).^14^ In this study, high dental anxiety (MDAS-N ≥19) was observed in 11 (3.54%) of the 311 participants. Other study showed 1 3% of patients in dental college and 20.90% of patients in dental clinics were severely anxious.^3^ One study reported that more than one third (36.9%) of the study population had DAS ≥15 (suggestive of highly anxious individuals).^15^ Another study done by telephone survey^4^ showed that 11.6% had high dental anxiety (DAS-N ≥19) while White, et al.^16^ reported 6.82% patients with high dental anxiety. In this study, females were more anxious than males. Though most studies,^5,6^ report females to be more anxious, a study in South India did not support for gender differences for anxiety.^7^

In the present study, 0-19 years age group had highest mean score of 12.43±3.830 (Table 3) similar to other study which showed 54.1% had severe dental anxiety,^17^ but Viinikangas, et al. study reported less score for older individuals.^18^ The prevalence of anxiety was seen mostly in the 20-30-year age group (37.3%).^19^ The anxiety level can be decreased by dental screening and education.^17^ Medical and dental students were less anxious than arts and computer science students suggesting role of dental education in reducing dental anxiety.^20,21^

In current study, participants were more anxious on the day before appointment than on the day during waiting for an appointment where as in Inamdar, et al. study, the level of anxiety while waiting for a dental treatment, increased a little as compared to the anxious anticipation of dental treatment a day earlier, approximately 1 0% of the individuals were more anxious.^15^ In this study, MDAS score had been found the highest with local anaesthesia and tooth drilling where similar study done on students reported that students were anxious about tooth drilling and local anaesthetic injection. While anticipating a dental treatment for the next day, 35.3% of participants were not anxious. There were various levels of anxiety among the remaining 65.7%. Total of 64% of these respondents were extremely anxious to slightly anxious. Anxiety levels increased further when participants were asked about getting their tooth drilled and 89% of the respondents were anxious about getting their tooth drilled.^15^ similarly, higher level of anxiety was found to be exhibited by participants for injection (55%), followed by scaling (45%) and tooth drilling (40%).^22^

Local anaesthesia is given for other dental procedure and related to dental delayed dental visit.^23^ High anxiety, younger age, and traumatic dental history were correlated with greater increases in heart rate during the administration of local dental anaesthesia.^24^ dental education, psychotherapy might be helpful in reducing anxiety regarding local anaesthesia.

The mean MDAS-N score was highest (11.83±3.774) among bachelors and above education group, but the difference was minimal (Table 4). This may be due to more awareness for oral health and it is multifactorial which is governed by social, cultural rituals and education. Anxiety is present in other patient category(dyspepsia and irritable bowel syndrome).^25^ No significant difference result have been found between death-related occupations (firemen, funeral personnel) and non-death related occupations (secretaries, accountants, teachers, etc.) measured by death related anxiety scale.^26^ Limitations of the study include singlecentre study with small sample size.

## CONCLUSIONS

The prevalence of anxiety among patients visiting fot periodontal therapy in this study was found to be higher compared to other study done in similar setting. Further studies with other modifying variables are needed to conclude this finding regarding dental anxiety in periodontal therapy and its reduction for patient comfort.
